# Increased expression of Ki-67 is a poor prognostic marker for colorectal cancer patients: a meta analysis

**DOI:** 10.1186/s12885-019-5324-y

**Published:** 2019-02-06

**Authors:** Zhao-Wen Luo, Ming-Gu Zhu, Zhi-Qiao Zhang, Feng-Jun Ye, Wen-Heng Huang, Xue-Zhang Luo

**Affiliations:** Department of Internal Medicine, The Affiliated Chencun Hospital of Shunde Hospital, Southern Medical University, Shunde, 528313 Guangdong China

## Abstract

**Background:**

The prognostic value of Ki-67 expression in colorectal cancer patients was controversial. Therefore, this meta analysis was conducted to ascertain the prognostic value of Ki-67 expression in colorectal cancer patients.

**Methods:**

The electronic databases, including EMBASE, PubMed, Cochrane Library and Web of Knowledge database, were searched from January 1970 to July 2017. The pooled hazard ratios and 95% confidence intervals were calculated to evaluate the prognostic value of Ki-67 expression for colorectal cancer patients.

**Results:**

Totally 34 eligible studies and 6180 colorectal cancer patients were included in the present meta analysis. The pooled hazard ratios were 1.54(95% CI 1.17–2.02, *P* = 0.005) for overall survival and 1.43(1.12–1.83, *P* = 0.008) for disease free survival in univariate analysis. After adjustment of other prognostic factors, the pooled HR was 1.50(95% CI 1.02–2.22, *P* = 0.03) for overall survival in multivariate analysis.

**Conclusion:**

The present meta analysis demonstrated that high Ki-67 expression is significantly correlated with poor overall survival and disease free survival, indicating that high Ki-67 expression may serve as a valuable predictive method for poor prognosis of colorectal cancer patients.

## Introduction

Colorectal carcinoma (CRC) is the fourth most common cause of death induced by carcinoma, accounting for 600,000 deaths per year [1]. Some clinical factors, such as tumor stage, tumor necrosis, vascular invasion and differentiation, have been reported to be associated with prognosis of CRC patients [2, 3]. However, these factors were not sufficient to accurately make risk stratification for clinical prognosis. In clinical practice, identification of patients with high risk of poor prognosis would help to optimize treatment and to improve clinical prognosis. Therefore, there is an urgent need to find reliable prognostic factors for prediction of clinical prognosis in CRC patients.

Ki-67 antigen expresses throughout the cell cycle in proliferating cells, except for quiescent (G0) cells [4]. The correlation between Ki-67 expression and prognosis of CRC patients were still contradictory in various studies [5–38]. Several studies showed that high Ki-67 expression was related with poor overall survival (OS) [26–30], whereas several studies reported that high Ki-67 expression was correlated with favorable OS [8, 15, 16]. Therefore, we performed this meta analysis to determine the prognostic value of Ki-67 expression in CRC patients.

## Materials and methods

### Literature search strategy

Four electronic databases, including EMBASE, PubMed, Web of Knowledge and Cochrane Library, were searched from January 1990 to July 2017 for eligible studies. We performed literature search by combined text word and MeSH strategy for PubMed with the following search strategy: (“Ki67” or “Ki-67” or “MIB-1” or “MIB1”) and (“carcinoma” or “neoplasm” or “tumor” or “cancer” or “malignancy”) and (“colorectal” or “rectal” or “colon”) and (“prognosis” or “prognostic” or “survival” or “outcome” or “mortality”).The strategies for EMBASE and other databases were similar but were adapted according to the guideline of the database. Meanwhile, expanded search of hyponym was performed. In the retrieval process, we made a manual search using the references of the including articles to supplement eligible studies. We contacted the corresponding author to get necessary study data if necessary. The search was restricted to human studies and there was no restriction in terms of language or publication time. All information investigation and collection were conducted according to the principles of Declaration of Helsinki.

### Criteria for inclusion and exclusion

The inclusion criteria were defined as follows: (1) proven pathological diagnosis of CRC in humans; (2) assessment of Ki-67 expression by using immunohistochemistry (IHC) method; (3) enough survival information such as hazard ratio (HR) and 95% confidence interval (CI). Studies not directly providing hazard ratio and 95% confidence interval were included if survival information were available from survival curves or tables. Articles published in Chinese were included in the present meta analysis as English literature. For multiple studies from the same study population, only the most recently published study was included in the present analysis.

The following studies were excluded: (1) non-human experiments; (2) case reports, reviews, letters and conference abstracts without survival information; (3) lack of the necessary survival information.

### Quality assessment

Two reviewers (Zhi-Qiao Zhang and Zhao-Wen Luo) independently assessed the quality of the studies included in the present meta analysis using Newcastle-Ottawa Quality Assessment Scale (NOS). The NOS comprises assessments of patient selection, study comparability, follow-up and outcomes of interest. The total scores were used to evaluate the quality of the study. Disagreements between two reviewers (Zhi-Qiao Zhang and Zhao-Wen Luo) were resolved by consultation with a third reviewer (Ming-Gu Zhu).

### Data extraction

Two investigators (Zhi-Qiao Zhang and Zhao-Wen Luo) independently extracted the survival information of the original studies: surname of the first author, country, study sample size, publication year, disease stage, test method of Ki-67 expression, clinical parameters, and survival information (HRs and 95%CIs). All study information were extracted and recorded by using a standardized form. All included studies were coded as the surname of the first author + publish year in the standardized form. Study authors were contacted to get necessary data if necessary. Disagreements between two investigators were resolved by discussion with a third investigator (Ming-Gu Zhu).

### Statistical analysis

The statistical analysis was performed according to the suggestions advised by the Meta analysis of Observational.

Studies in Epidemiology group(MOOSE) [39]. The hazard ratios (HR) and 95% CI were used to summary the final effects of survival outcome. We pooled HR and 95% CI if the survival data were reported in the original articles. While HR and 95% CI were not directly reported in the articles, survival data was extracted from Kaplan-Meier curve and used to estimate HR. The heterogeneity among various studies was measured by the Q and *I*^2^ tests. A probability value of *I*^2^ value≥30% and *P* value< 0.1 indicated the existence of significant heterogeneity. A random effect model (DerSimonian and Laird method) or fixed effect model(Mantel-Haenszel method) was used according to the results of heterogeneity analyses. Meta-regression analyses (Reml method) and subgroup analyses were performed to explore the sources of heterogeneity. Funnel plot, Begg’s test, Egger’s test, and trim and fill method were performed to assess publication bias. The difference was considered to be statistically significant if *P* value < 0.05. The statistical analyses were performed by STATA version 12.0 software (Stata Corporation, College Station, Texas, USA).

## Results

### Search results

**Fig. 1:**
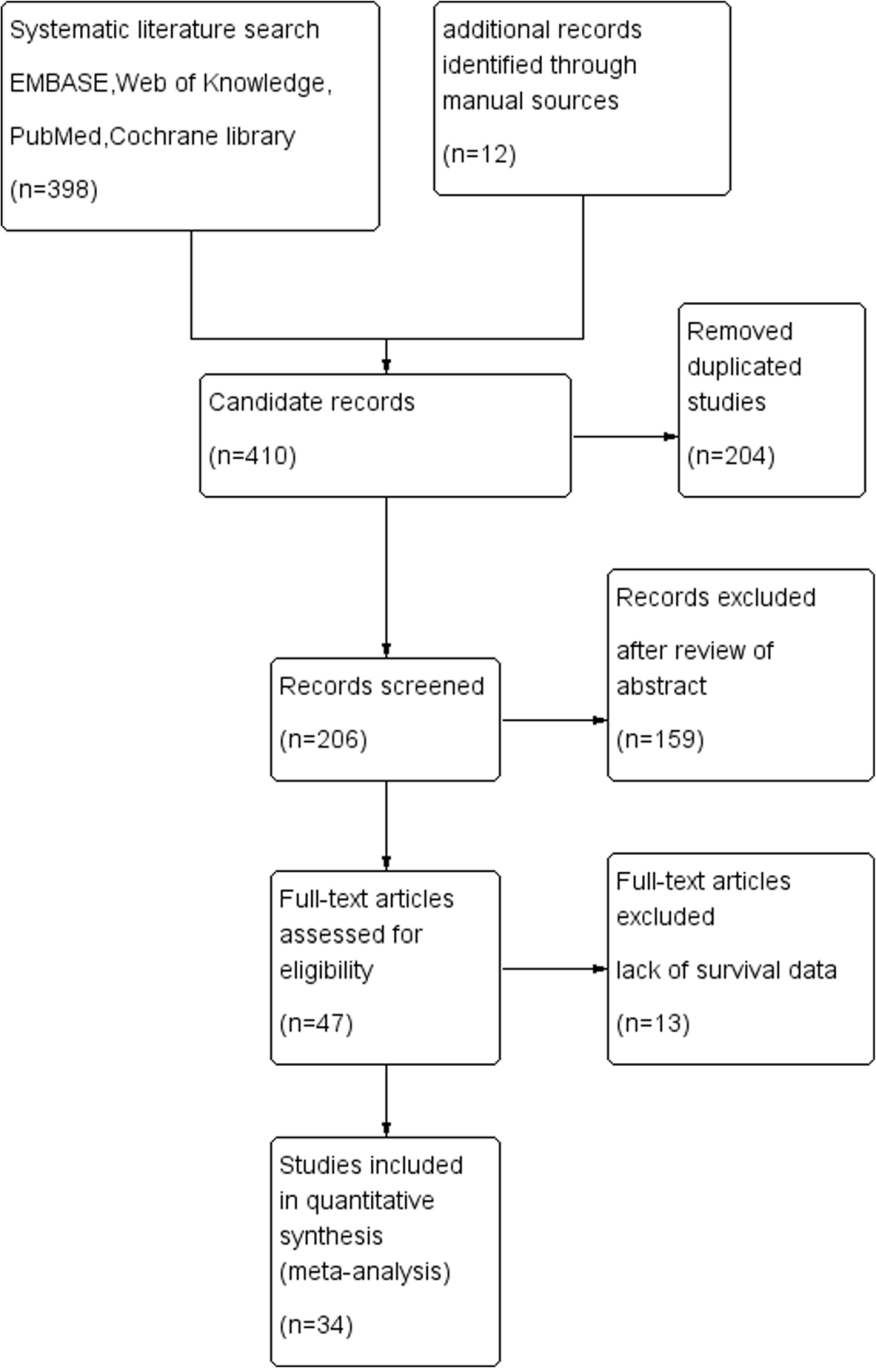
Flowchart of study selection in the present meta analysis

The initial search returned a total of 410 articles (with 204 duplicate articles). After screening the abstracts, 159 irrelevant articles were excluded according to the criteria for inclusion and exclusion. Reviewers identified 47 potential studies for full-text review and 13 articles were eliminated due to inadequate data for meta analysis. Finally, a total of 34 studies were included in the present meta analysis [5–38]. The details of screening process were shown in Fig. 1. Quality assessment of 34 eligible studies were performed by using the Newcastle-Ottawa Scale (NOS).

### Study selection and characteristics

The characteristic of the 34 included studies were summarized in Table 1. The publication time ranged from 1997 to 2015.The patient number of 34 studies ranged from 30 to 867,with a mean sample size of 182. The mean length of follow-up period ranged from 23 to 130 months. The NOS scores for quality of 34 studies in this meta analysis varied from 6 to 7, with a mean value of 6.5. Ki-67 expression were measured in surgical tumor tissues.

**Table 1:** Characteristics of studies included in the present meta analysis

Author/Country/Year	Detection	Quantitative	Antibody	Fixed	Positive^a^	Median age	History		Cut off	Patient
	method	method	type	method	Rate(%)	(Range)	Type/ Stage	Therapy	Value	Number
Meyer [5] Germany 2009	microscope	semi-quantitative	mouse monoclonal	formalin	88.8%	64(28–91)	CC II-IV	S + C	NR	392
Garrity [6] Canada 2004	NR	quantitative	NR	formalin	62.4%	NR	CRC II-III	S + C	27.0%	366
Fodor [7] America 2012	Tissue microarrays	quantitative	NR	formalin	52.0%	71.5(18–97)	CRC I-IV	S + C	60.0%	867
Salminen [8] Finland 2005	microscope	quantitative	mouse monoclonal	formalin	45.2%	66(36–86)	RC I-IV	S + R	40.0%	146
Zhang [9] China 2015	Tissue microarrays	quantitative	NR	formalin	47.9%	72(37–93)	CRC I-IV	S + R	40.0%	73
Yoshimura [10] Japan 2003	microscope	quantitative	monoclonal	NR	NR	65	CRC III	S	62.0%	56
Li [11] China 2010	Tissue microarrays	semi-quantitative	monoclonal	formalin	78.8%	65(22–95)	CC I-IV	S	10.0%	203
Li [12] China 2011	Tissue microarrays	semi-quantitative	monoclonal	formalin	78.8%	65(22–95)	CC I-IV	S	10.0%	203
Dawson [13] Switzerland 2014	Tissue microarrays	quantitative	monoclonal	formalin	NR	71(35–93)	CRC I-IV	S + C	30.0%	188
Weber 2001 [14] France 2001	microscope	quantitative	mouse monoclonal	formalin	50.2%	61(29–80)	CRCLM I-IV	S + C	50.0%	221
Xi [15] China 2011	microscope	semi-quantitative	mouse monoclonal	formalin	59.7%	62(20–81)	CRC I-IV	S	score > 5	201
Ivanecz [16] Slovenia 2014	microscope	semi-quantitative	mouse monoclonal	formalin	28.0%	62(27–78)	CRCLM I-IV	S + C	50.0%	98
Rosati [17] Italy 2004	microscope	semi-quantitative	monoclonal	formalin	55.3%	66(29–79)	CRC II-III	S + C	10.0%	103
Nishihara [18] Japan 2009	microscope	quantitative	mouse monoclonal	formalin	51.2%	68(33–94)	CRC I-IV	S	40.0%	201
Scopa [19] Greece 2003	microscope	semi-quantitative	mouse monoclonal	NR	64.0%	66(25–82)	CRC I-IV	S + C	5.0%	117
Visca [20] Italy 1999	microscope	semi-quantitative	mouse monoclonal	formalin	90%	64	CRC I-IV	S	20.0%	100
Handa [21] Japan 1999	microscope	quantitative	monoclonal	formalin	65.8%	66(39–90)	CRC I-IV	S	19.5%	73
Bahnassy [22] Egypt 2004	microscope	quantitative	NR	NR	NR	NR	CRC I-IV	S	11.5%	60
Buglioni 1999 [23]	microscope	quantitative	NR	NR	50.3%	64(56–70)	CRC I-IV	S	25.0%	171
Bertolini [24] Italy 2007	microscope	quantitative	NR	formalin	90.0%	64(26–78)	RC I-III	S + C + R	20.0%	91
Kimura [25] Japan 2000	microscope	quantitative	monoclonal	formalin	31.0%	63.1 ± 10.7	CRC II-IV	S	50.6%	71
Fernandez-Cebrian [26] Spain 2007	microscope	semi-quantitative	mouse monoclonal	NR	69.1%	63.9(45–96)	CC II	S	25.0%	162
Wu 2012 [27] China 2012	microscope	semi-quantitative	mouse monoclonal	formalin	78.7%	62(22–83)	CRC I-IV	S	score > 5	192
Furudoi 2001 [28] Japan 2001	microscope	quantitative	monoclonal	formalin	45.0%	63.2 ± 13.9	CRC II-IV	S + C	47.9%	111
Lin [29] China 2008	microscope	semi-quantitative	mouse monoclonal	NR	18.3%	64	CRC I-IV	S	50.0%	60
Hayashi 2015 [30] Japan 2015	microscope	quantitative	mouse monoclonal	formalin	63.5%	65(35–83)	CRCLM I-IV	S + C	30.0%	54
Ajani [31] America 2010	microscope	quantitative	monoclonal	formalin	50.0%	NR	RC I-IV	C + R	median	30
Li [32] China 2009	microscope	quantitative	monoclonal	formalin	78.8%	65(22–95)	CC I-IV	S	10.0%	203
Hu 2009 [33] China 2009	microscope	quantitative	NR	formalin	30.7%	56.6(34–75)	CRC NR	S	10.0%	52
Roxburgh [34] Scotland 2013	Tissue microarrays	quantitative	mouse monoclonal	NR	61%	NR	CRC I-III	S + C	15%	230
Shin 2014 [35] Korea 2014	microscope	quantitative	NR	NR	74.4%	63(30–87)	CRC I-IV	S + C	50.0%	266
Nash [36] America 2010	microscope	quantitative	NR	formalin	66.0%	63(19–84)	CRCLM I-IV	S + C	50.0%	188
Prall [37] Germany 2004	Tissue microarrays	quantitative	mouse monoclonal	NR	50.0%	64.8(29–90)	CRC I-IV	S + C + R	median	149
Zlobec [38] Switzerland 2008	Tissue microarrays	quantitative	NR	NR	42.5%	68.7(36–96)	RC I-IV	S + C + R	15.0%	482

*NR* not reported, *OS* overall survival, *DFS* disease free survival, *S* surgery, *C* chemotherapy, *R* radiotherapy, *CRC* colorectal cancer, *CRCLM* colorectal liver metastases, *CC* colon cancer, *RC* rectal cancer, *NOS* Newcastle-Ottawa Quality Assessment Scale, *IHC* immunohistochemistry

^a^positive status was defined according to cut off value

### Prognostic value of high Ki-67 expression

**Fig. 2:**
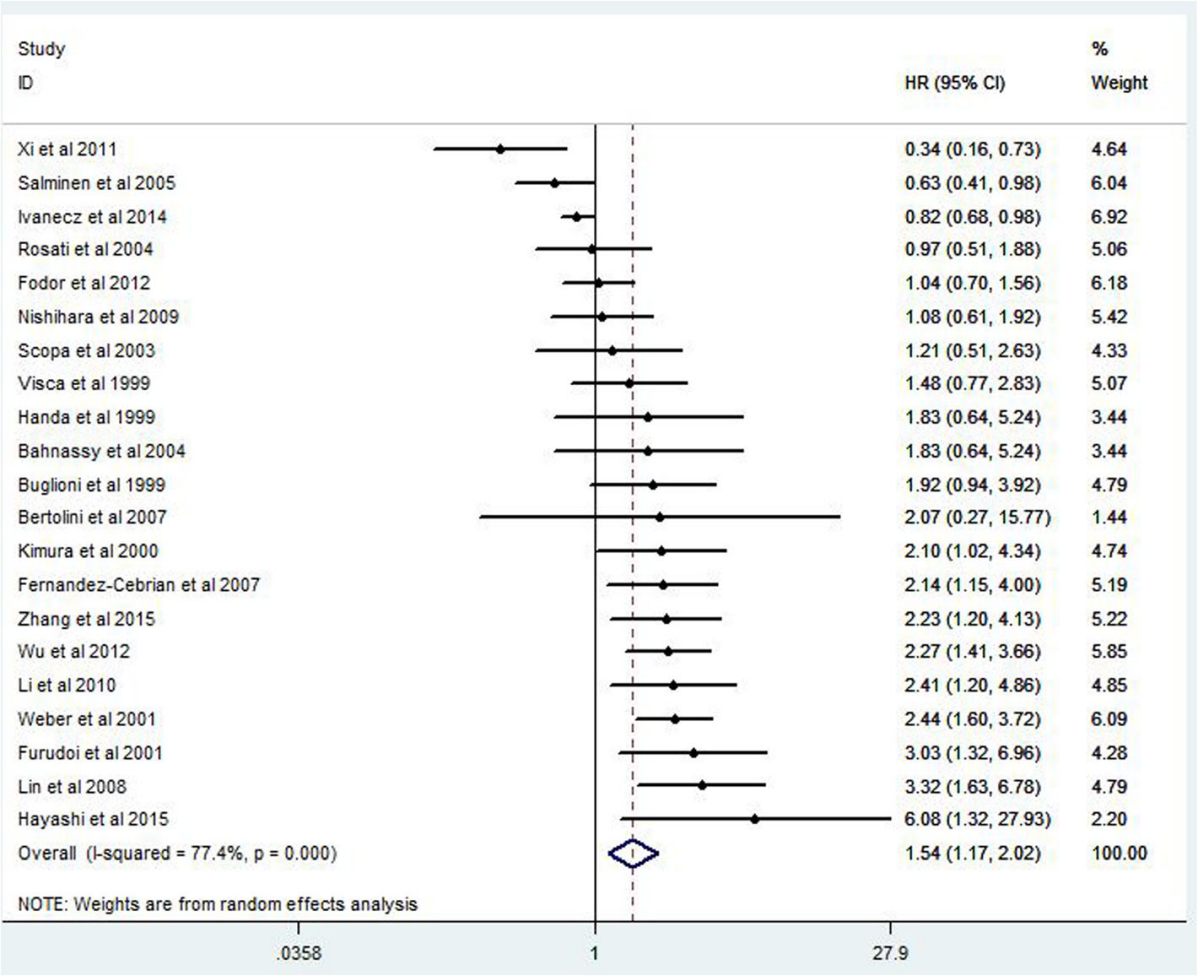
Forest plot diagrams of Ki-67 expression for overall survival in univariate analysis

**Fig. 3:**
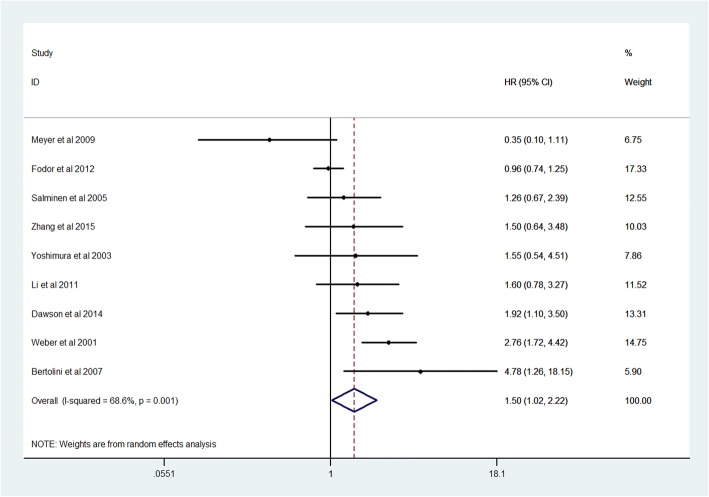
Forest plot diagrams of Ki-67 expression for overall survival in multivariate analysis

**Fig. 4:**
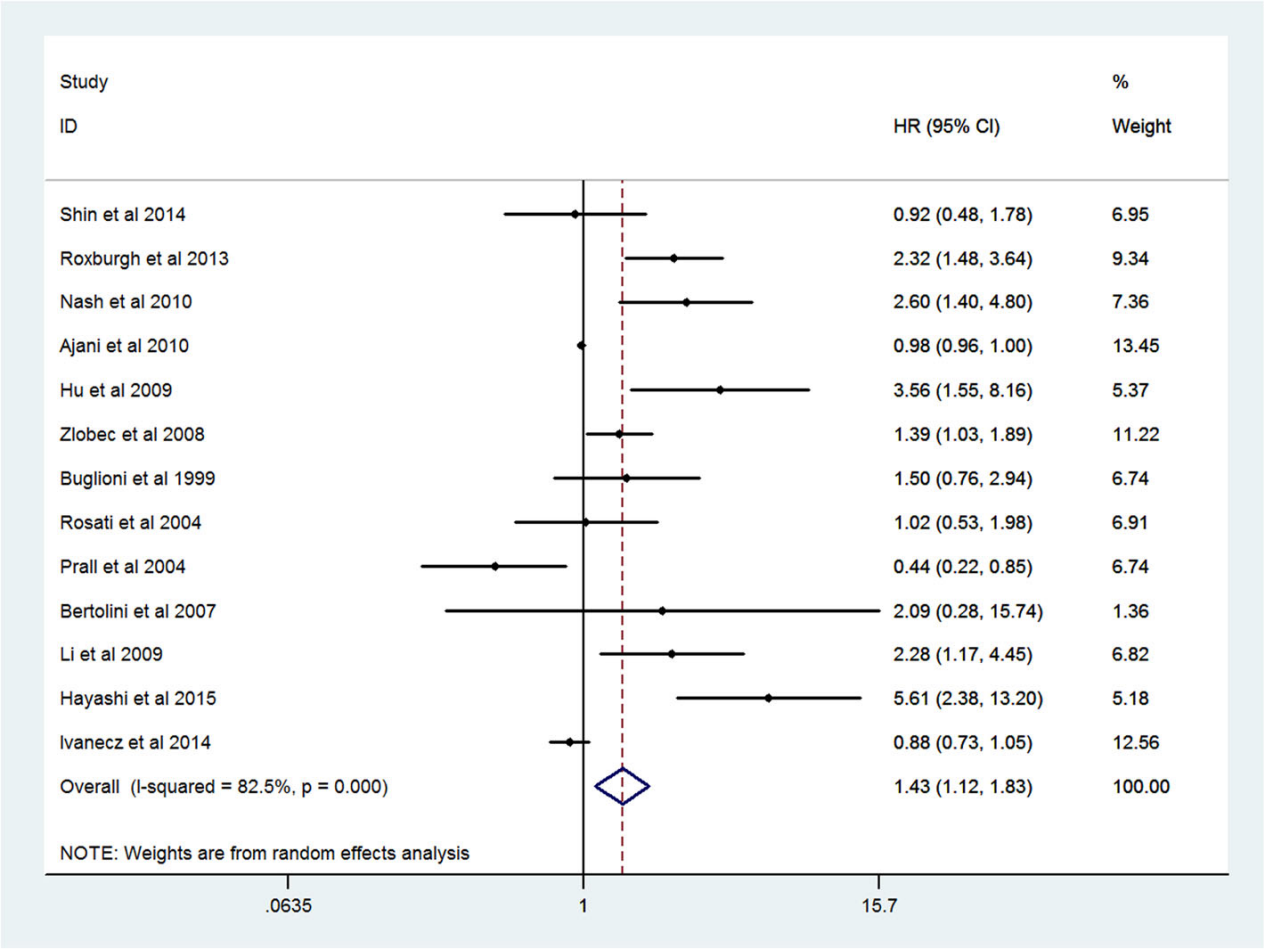
Forest plot diagrams of Ki-67 expression for disease free survival in univariate analysis

Finally 34 studies and 6180 CRC patients were collected and analyzed for prognostic value of Ki-67 expression. The pooled HR was 1.54(95% CI 1.17–2.02, *P* = 0.005) for overall survival (OS) in univariate analysis (Fig. 2). After adjustment of other prognostic factors, the pooled HR was 1.50(95% CI 1.02–2.22, *P* = 0.03) for OS in multivariate analysis (Fig. 3). The pooled HR was 1.43(95% CI 1.12–1.83, *P* = 0.008) for disease survival (DFS) in univariate analysis (Fig. 4).

### Publication bias

**Fig. 5:**
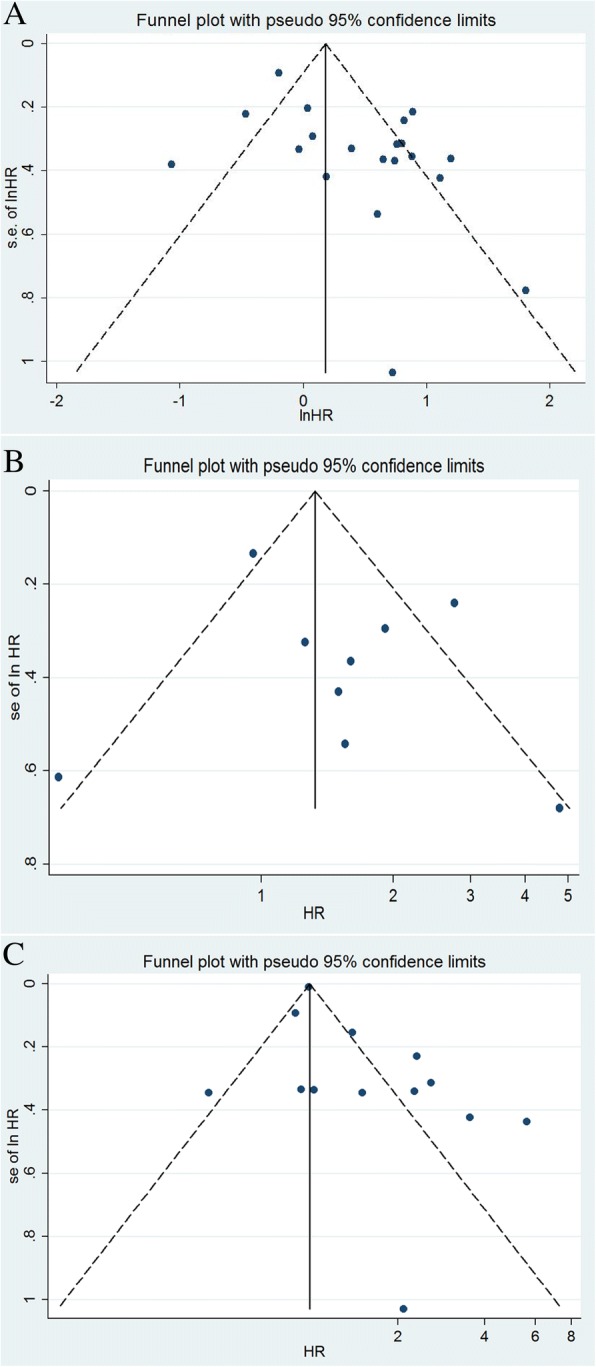
Funnel plot of high Ki-67 expression. **a** Overall survival in univariate analysis; **b** Overall survival in multivariate analysis; **c** Disease free survival in univariate analysis

For OS in univariate analysis, the funnel plot of Ki-67 expression showed obvious asymmetry (Fig. 5a), indicating that the pooled results might be influenced by the publication bias. The Begg’s test and Egger’s test were further performed to explore publication bias in the present meta analysis. There was significant publication bias according to Egger’s test (*P* = 0.008) whereas Begg’s test did not show significant publication bias (*P* = 0.629).

For OS in multivariate analysis, the funnel plot of Ki-67 expression did not show obvious asymmetry (Fig. 5b). There was no significant publication bias according to Egger’s test (*P* = 0.378) and Begg’s test (*P* = 1.0).

For DFS in univariate analysis, the funnel plot of Ki-67 expression showed obvious asymmetry (Fig. 5c). There was significant publication bias according to Egger’s test (*P* = 0.032) whereas Begg’s test did not show significant publication bias in the present study (*P* = 0.246).

### Contour-enhanced funnel plot

The contour-enhanced funnel plot can help to ascertain whether funnel plot asymmetry due to publication bias [40]. Contour lines which indicated conventional milestones in levels of statistical significance (0.05, and 0.1) are added to funnel plots. If the dummy studies lie in areas of statistical nonsignificance, then this provides evidence of the possibility that the funnel plot asymmetry is due to publication bias. Conversely, if the dummy studies are in areas of high statistical significance(*P* > 0.05), this will support that the cause of the funnel plot asymmetry may be caused by factors other than publication bias, such as poor methodological quality or true heterogeneity.

**Fig. 6:**
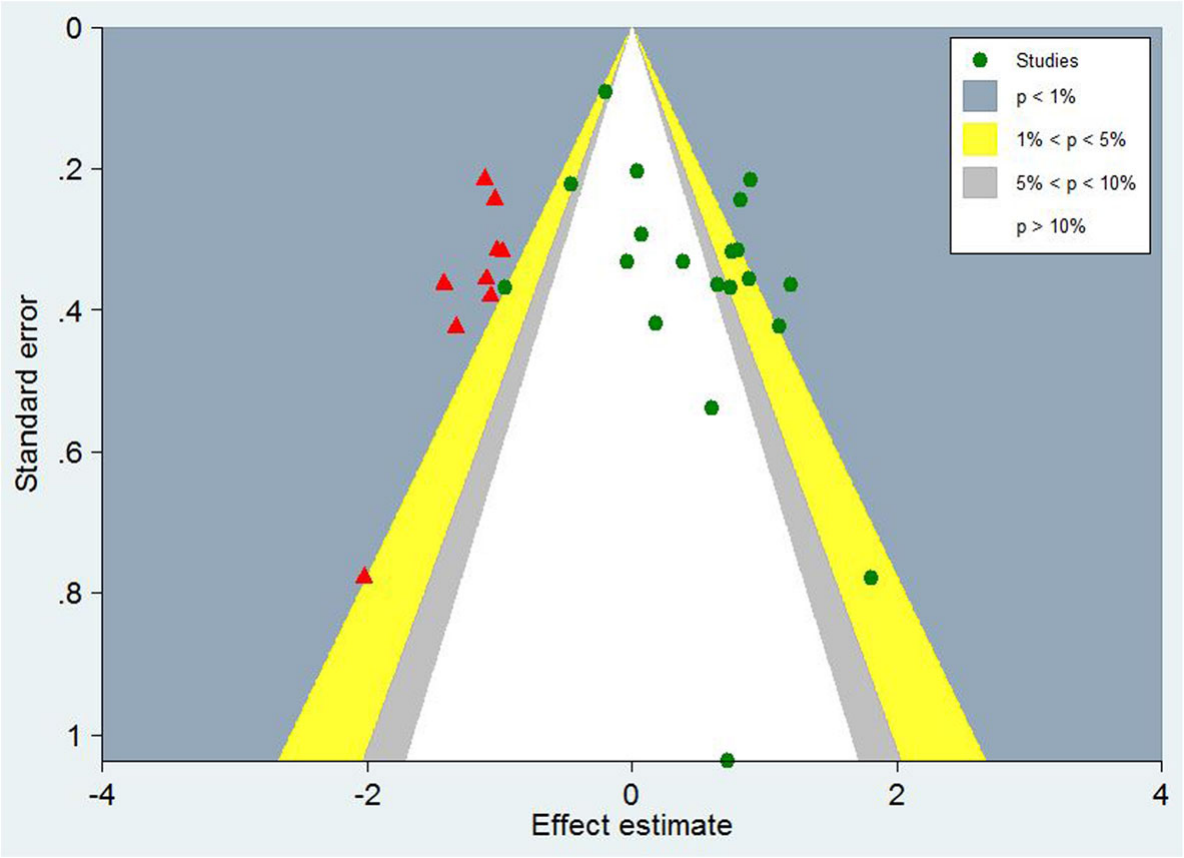
Contour-enhanced funnel plot for overall survival in univariate analysis

To explore the potential causes of funnel plot asymmetry for OS in univariate analysis, a contour-enhanced funnel plot by using the trim-and-fill method was performed. The dummy studies were indicated by red triangles and the genuine studies were indicated by green dots (Fig. 6). This trim-and-fill method added 9 dummy studies to balance the funnel plot and all 9 dummy studies were in areas of high statistical significance, suggesting that the publication bias might not the major cause of funnel plot asymmetry in the present meta analysis.

### Meta-regression analysis and subgroup analysis

Subgroup analysis (Table 2) suggested that cut off value, treatment and pathologic type might contribute to the clinical heterogeneity. The pooled HRs were 0.743 (95% CI 0.332–1.662, *I*^2^ = 20.7%, *P* = 0.261) for rectal cancer (RC) patients and 1.902(0.695–5.198, *I*^2^ = 92%, *P* = 0.001) for colorectal liver metastases (CRCLM) patients. Further meta- regression analysis did not find any significant source of heterogeneity (all *P* > 0.05).

**Table 2:** Subgroup analysis for association between Ki-67 expression and overall survival

Term	Subgroup	Number	Pooled	95%CI	*P*	Heterogeneity	*P*
		of study	HR			*I* ^*2*^	
Location	Asian	10	1.89	1.23–2.90	0.003	70.9	0.001
	Non- Asian	11	1.282	0.936–1.758	0.122	77.4	0.001
Cut off value	≥40%	9	1.5	1.01–2.24	0.044	84.9	0.001
	<40%	10	1.71	1.33–2.21	0.001	0	0.529
Patient number	≥100	13	1.381	0.994–1.918	0.054	75.7	0.001
	<100	8	2.008	1.131–3.564	0.017	79.4	0.001
Positive rate^a^	≥50%	14	1.513	1.119–2.046	0.007	64.2	0.001
	<50%	6	1.589	0.911–2.773	0.103	86.5	0.001
Pathologic type	CRC	14	1.526	1.135–2.05	0.005	62.8	0.001
	CC	2	2.257	1.418–3.594	0.001	77.7	0.001
	RC	2	0.743	0.332–1.662	0.469	20.7	0.261
	CRCLM	3	1.902	0.696–5.198	0.21	92.6	0.001
Treatment	S	12	1.518	1.038–2.221	0.031	74.6	0.001
	S + C	7	1.477	0.933–2.338	0.096	82.3	0.001
	S + C + R	2	2.216	1.227–4.002	0.008	0	0.945
Extraction method	Curve	7	1.261	0.915–1.736	0.001	57.3	0.029
	Reported	14	2.095	1.491–2.02	0.156	72.5	0.001

*HR* hazard ratio, *CI* confidence interval, *CRC* colorectal cancer, *CRCLM* colorectal liver metastases, *CC* colon cancer, *RC* rectal cancer, *S* surgery, *C* chemotherapy, *R* radiotherapy

^a^positive status was defined according to cut off value

**Fig. 7:**
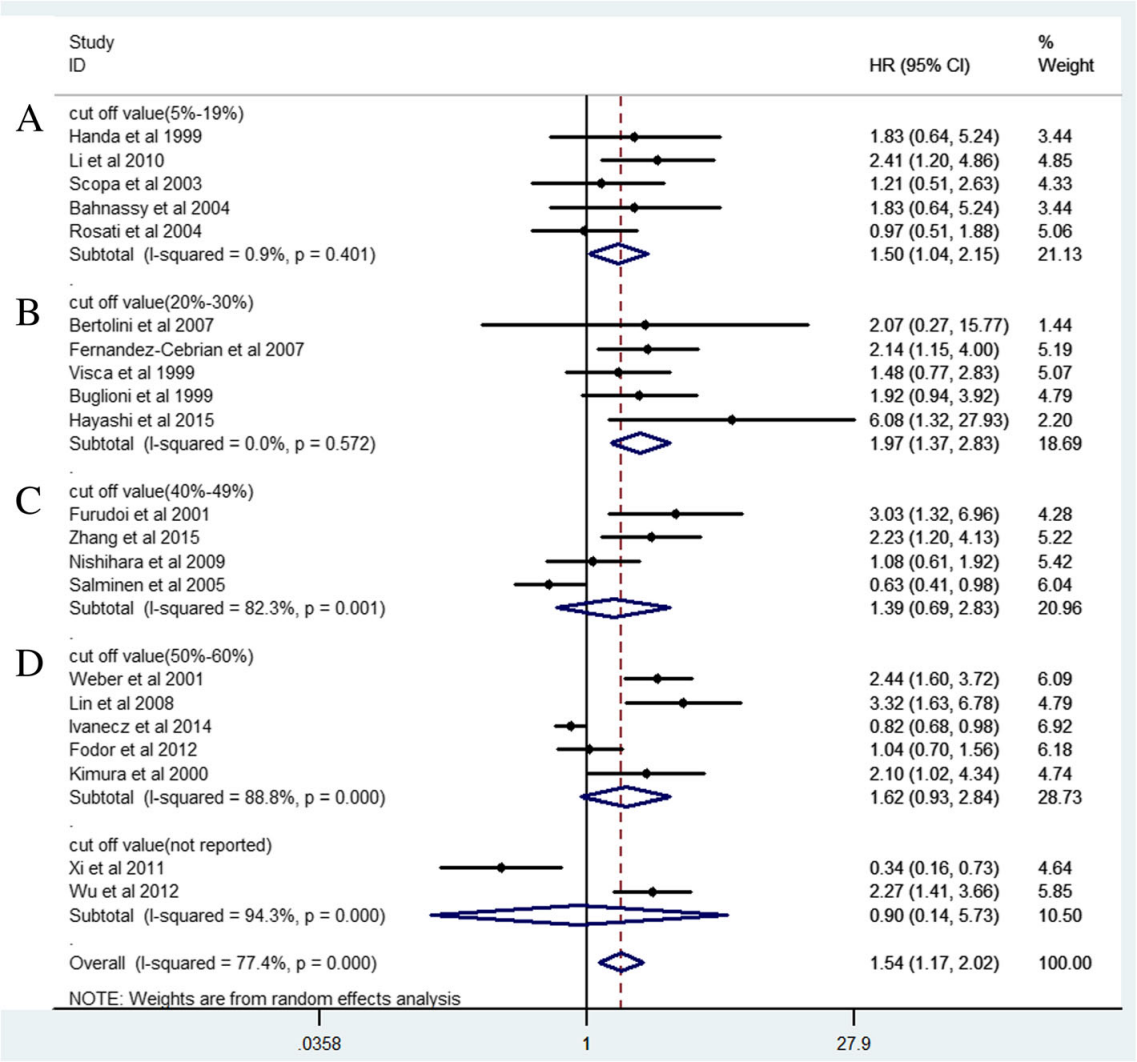
Forest plot diagrams of Ki-67 expression for overall survival according different cut off values. **a** Cut off value (5–19%); **b** Cut off value (20–30%); **c** Cut off value (40–49%); **d** Cut off value (50–60%)

According to the results of subgroup analysis, we further explored the association between Ki-67 expression and overall survival with different cut off values (Fig. 7). The pooled HR (1.97, 95% CI 1.37–2.83) in group with cut off value (20–30%) was significantly higher than that in other groups, indicating that cut off value (20–30%) might be more suitable for clinical practice than other cut off values.

### Sensitivity analyses

All studies were sequentially removed to explore that whether any individual study had a significant influence to the pooled HRs. The pooled HRs of sensitivity meta analysis ranged from 1.49(95%CI: 1.13–1.96) to 1.65 (95%CI: 1.26–2.15) for OS in univariate analysis, demonstrating that the pooled HRs were not significantly affected by any individual study (Table 3).

**Table 3:** Effect of individual studies on the pooled hazard ratios of Ki-67 expression for overall survival

Study omitted	HR	Lower value of 95% CI	Upper value of 95% CI
1	1.6470636	1.2618686	2.1498425
2	1.6286341	1.2338984	2.1496494
3	1.6086709	1.2262427	2.110367
4	1.5804045	1.1893544	2.1000286
5	1.5853532	1.1829669	2.1246113
6	1.5744233	1.1822454	2.0966956
7	1.5586835	1.1753646	2.0670133
8	1.5452128	1.1628222	2.0533514
9	1.5309644	1.158212	2.0236814
10	1.5309644	1.158212	2.0236814
11	1.5234994	1.150002	2.0183012
12	1.5333095	1.1640916	2.0196332
13	1.5163125	1.145869	2.0065153
14	1.5121837	1.1424872	2.00151
15	1.5081896	1.1403373	1.9947045
16	1.5015592	1.136433	1.9839972
17	1.5038622	1.1387317	1.9860705
18	1.4894764	1.1326977	1.9586338
19	1.4916791	1.1332084	1.9635456
20	1.4770394	1.1257362	1.9379722
21	1.4908768	1.1371239	1.9546804
Combined	1.5392004	1.1725948	2.0204233

*HR* hazard ratio, *CI* confidence interval

### Cumulative meta analysis of Ki-67 expression

**Fig. 8:**
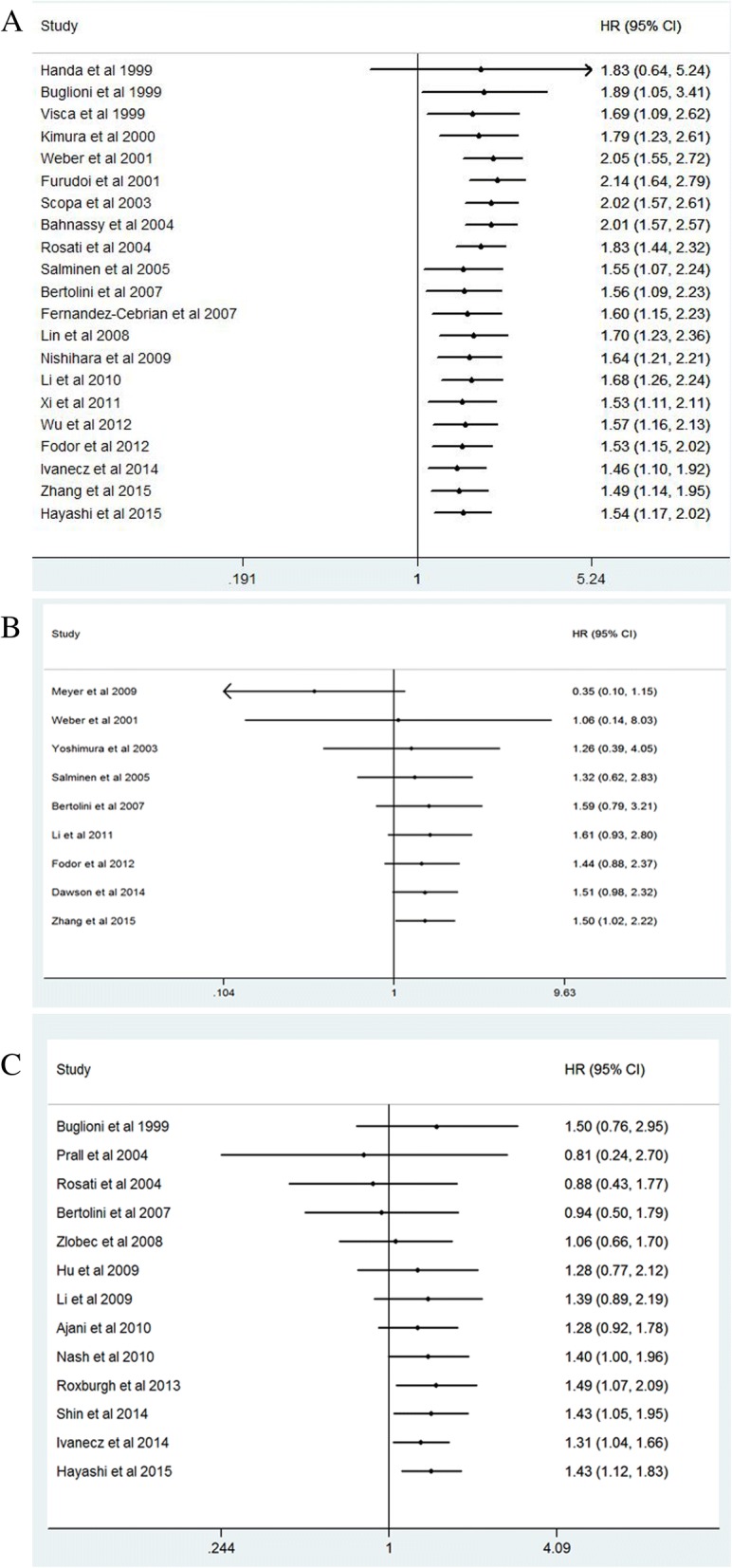
Cumulative meta analysis of Ki-67 expression. **a** Overall survival in univariate analysis; **b** Overall survival in multivariate analysis; **c** Disease free survival in univariate analysis

We further performed cumulative meta analysis to assess the stability of Ki-67 expression for OS in univariate analysis (Fig. 8a), OS in multivariate analysis (Fig. 8b) and DFS in univariate analysis (Fig. 8c). The pooled HRs of cumulative meta analysis ranged from 1.48(95%CI 1.1–2.0) to 2.14 (95%CI 1.64–2.79) for OS in univariate analysis since 1999, demonstrating that performance of Ki-67 expression for OS in CRC patients was stable and reliable.

## Discussion

The present meta analysis showed that high Ki-67 expression is significantly correlated with poor prognosis in CRC patients. The pooled hazard ratios were 1.54(95% CI 1.17–2.02, *P* = 0.005) for overall survival and 1.43(1.12–1.83, *P* = 0.008) for disease free survival in univariate analysis. After adjustment of other prognostic factors, the pooled HR was 1.50(95% CI 1.02–2.22, *P* = 0.03) for overall survival in multivariate analysis.

Some previous studies have reported that high Ki-67 expression significantly predicted poor OS in CRC patients [26–30]. The conclusion of the present meta analysis was consistent with that of these previous studies. Although the mechanism of Ki-67 expression on tumor prognosis was still uncertain, some clinical studies have provided interesting evidences for utility of Ki-67 expression as a predictive tool for clinical prognosis in patients with different tumors, for example prostate cancer, thyroid carcinoma and renal clear cell [41–43]. Interestingly, there were three studies indicated that there was a negative association between the high Ki-67 expression and the overall survival of CRC patients. Salminen et al. [8] reported that HR of high Ki-67 expression was 0.63 (95% CI 0.41–0.98) for overall survival in univariate analysis, whereas it was 1.26 (95% CI 0.67–2.39) for overall survival in multivariate analysis. Xi et al. [15] reported that HR of high Ki-67 expression was 0.34 (95% CI 0.16–0.73) for overall survival by using a special scoring system of Ki-67 expression: The intensity of positivity was scored as follows: 0, negative; 1, weak; 2, moderate; 3, strong. The extent of positivity was scored according to the percentage of cells showing positive staining: 0, < 5%; 1, 5–25%; 2, 25–50%; 3, 50–75%; 4, > 75%. The final score was determined by multiplying the intensity of positivity and the extent of positivity scores and final score > 5 was defined as positive. Ivanecz et al. [16] selected 98 patients for prognostic study from 406 patients by using the following criteria: first liver resection for CLM; potentially curative R0 resection; follow-up period of surviving patients > 5 years. The special inclusion criteria might influence the baseline characteristics and weaken the credibility of study conclusion.

There was significant heterogeneity in the present meta analysis. The current meta analysis might exist several potential sources of heterogeneity as follows: First, the heterogeneity caused by different cut off values of Ki-67 expression was inevitable. The subgroup analysis have revealed the influence of different cut off values on heterogeneity. Second, the subgroup analysis suggested that different treatment has a significant effect on heterogeneity and might be a potential source of heterogeneity. Third, obvious difference of the pooled HRs between rectal cancer subgroup and colorectal liver metastases subgroup suggested that further study is need to clarify the exact influence of different pathologic type on clinical prognosis.

Publication bias is an important influence factor for interpreting the conclusion. The funnel plot for OS in univariate analysis was obviously asymmetrical and Egger’s test also demonstrated significant publication bias (*P* = 0.008). The funnel plot asymmetry might be caused by different reasons as follows: Firstly, publication bias was one important source of funnel plot asymmetry, for example location bias, citation bias, language bias, time lag bias, multiple publication bias and outcome reporting bias. Secondly, the true heterogeneity in intervention effects might be one resource of funnel plot asymmetry. The true heterogeneity might be caused by differences in the intensity of interventions or differences in underlying risk between studies with different sizes. Thirdly, poor methodological quality was another source of funnel plot asymmetry. Smaller studies were often performed with poorer methodological quality than larger studies. Therefore, smaller studies with poorer methodological quality also tended to display larger influences than larger studies. Finally, funnel plot asymmetrical might be caused by chance. We conducted a contour-enhanced funnel plot using the trim-and-fill method to explore the potential cause of funnel plot asymmetry. Given that all 9 dummy studies were in areas of high statistical significance, the publication bias might not the major cause of funnel plot asymmetry in the present meta analysis.

Although significant heterogeneity existed in the present meta analysis, sensitivity analysis demonstrated that the relation between high Ki-67 expression and poor prognosis of CRC patients did not changed after removing any individual study. Meanwhile, cumulative meta analysis also showed that performance of high Ki-67 expression for poor prognosis in CRC patients was stable and reliable.

The present meta analysis had several strengths: Firstly, there were 34 eligible studies and 6180 CRC patients in the present study, which could significantly increase persuasiveness of the conclusion. Secondly, Ki-67 expression in 34 eligible studies was all measured with IHC method, leading to less clinical heterogeneity. Thirdly, studies published in Chinese were also included in this meta analysis as English literature, increasing the representation of the study population.

The conclusion of the present meta analysis should be interpreted cautiously for reasons as follows: Firstly, most studies defined positive status of Ki-67 expression according to different cut off values, which might result in clinical heterogeneity. Hence, it is necessary to determine an universal cut off value for future studies. Secondly, heterogeneity was inevitable due to different baseline characteristics, such as tumor stage and treatment, which might increase clinical heterogeneity and reduce the persuasiveness of the conclusion. Thirdly, some survival data were extracted from survival curves indirectly. Although the method for extracting HR and 95% CI from survival curves is widely accepted, we could not completely eliminate the sources of information inaccuracy in the process of information extraction. Fourthly, Ki-67 expression in the selected studies was measured by IHC method. This method might suffer from important variation among different studies and was not enough precise for a rigorous and precise conclusion. Fifthly, several genes have been reported to be related with the prognosis of CRC patients including KRAS, Cyclin-dependent kinase 2 (cdc2), and Cathepsin D [5, 35, 36]. Interestingly enough, Nash reported that patients with KRAS mutant tumors that expressed high levels of Ki-67 had significantly worse DFS than patients with KRAS wild-type tumors that expressed low level of Ki-67, indicating that combined application of these predictive genes might provide more valuable prognostic information and further improve the clinical prediction of prognosis for CRC patients.

In conclusion, the present meta analysis demonstrated that high Ki-67 expression significantly predicts poor overall survival and disease free survival. High Ki-67 expression may serve as a valuable predictive biomarker for poor prognosis in CRC patients.

## Abbreviations

C: Chemotherapy
CC: Colon cancer
CI: Confidence interval
CRC: Colorectal cancer
CRCLM: Colorectal liver metastases
DFS: Disease free survival
HR: Hazard ratio
IHC: Immunohistochemistry
NOS: Newcastle-Ottawa Quality Assessment Scale
NR: Not reported
OR: Odds ratio
OS: Overall survival
R: Radiotherapy
RC: Rectal cancer
S: Surgery
